# Novel Organochlorinated Xerogels: From Microporous Materials to Ordered Domains

**DOI:** 10.3390/polym13091415

**Published:** 2021-04-27

**Authors:** Guillermo Cruz-Quesada, Maialen Espinal-Viguri, María Victoria López-Ramón, Julián J. Garrido

**Affiliations:** 1 Departamento de Ciencias, Edif. Los Acebos, Campus Arrosadía, Public University of Navarre, 31006 Pamplona, Spain; guillermo.cruz@unavarra.es; 2 Institute for Advanced Materials and Mathematics, Edif. Jerónimo de Ayanz, Campus Arrosadía, Public University of Navarre, 31006 Pamplona, Spain; 3 Departamento de Química Inorgánica y Orgánica; Facultad de Ciencias Experimentales, University of Jaén, 23071 Jaen, Spain; mvlro@ujaen.es

**Keywords:** xerogels, hybrid materials, TEOS, chloroalkyltriethoxysilane, inductive effect, textural properties, ORMOSILs

## Abstract

Hybrid silica xerogels combine the properties of organic and inorganic components in the same material, making them highly promising and versatile candidates for multiple applications. They can be tailored for specific purposes through chemical modifications, and the consequent changes in their structures warrant in-depth investigation. We describe the synthesis of three new series of organochlorinated xerogels prepared by co-condensation of tetraethyl orthosilicate (TEOS) and chloroalkyltriethoxysilane (ClRTEOS; R = methyl [M], ethyl [E], or propyl [P]) at different molar ratios. The influence of the precursors on the morphological and textural properties of the xerogels was studied using ^29^Si NMR (Nuclear Magnetic Resonance), FTIR (Fourier-Transform Infrared Spectroscopy), N_2_, and CO_2_ adsorption, XRD (X-ray Diffraction), and FE-SEM (Field-Emission Scanning Electron Microscopy). The structure and morphology of these materials are closely related to the nature and amount of the precursor, and their microporosity increases proportionally to the molar percentage of ClRTEOS. In addition, the influence of the chlorine atom was investigated through comparison with their non-chlorinated analogues (RTEOS, R = M, E, or P) prepared in previous studies. The results showed that a smaller amount of precursor was needed to detect ordered domains (ladders and T_8_ cages) in the local structure. The possibility of coupling self-organization with tailored porosity opens the way to novel applications for this type of organically modified silicates.

## 1. Introduction

The demand for materials with specific physicochemical properties has markedly increased over the past decade. Research has focused on the design of hybrid solids in which organic and inorganic species coexist on a nanometric scale, with the aim of generating synergistic effects and tailored materials. Organically modified silicates (ORMOSILs) are hybrid silicon xerogels that combine the mechanical, thermal, and structural rigidity and stability of inorganic materials with the flexibility and functionality of organic molecules [1]. These properties favor their utilization as chemical and optical sensors [2,3,4,5,6,7], catalysts [8,9], coatings [10,11,12,13,14], chromatographic agents [15,16], nanoprobes, and photovoltaic cells [17,18], among others.

The most widely adopted approach to hybrid xerogel synthesis is the sol–gel method [19], using either (i) “silane coupling agents” to provide a functional surface group that can be substituted or act as a bridge to other structural units [3,10,11,15] or (ii) co-condensation reactions or condensation between monomers of silicon tetraalkoxides (e.g., tetramethoxy- or tetraethoxysilane (TMOS, TEOS)) and one or more mono-, di-, or tri-alkylalkoxysilanes (R_x_Si(OR’)_4-x_). This procedure yields hybrid silicon materials based on a silica matrix with organic groups that modify the network, permitting the production of materials with specifically designed chemical and textural properties in a single process [20,21,22]. The incorporation of organic groups is known to promote the formation of ordered domains induced by intermolecular forces (e.g., hydrogen bonds, hydrophobic or electrostatic interactions, or π–π stacking) [23]. Although these forces may be weak at molecular level, they are capable of directing the nanostructuring mechanism at macromolecular level. This approach may offer a simple and efficient option for the preparation of transparent materials with controlled structure and porosity that can also have different morphologies (membranes, monoliths, and fibers, among others) [24]. The structure and texture of materials produced by the sol–gel method are known to be influenced by the molar percentage of R_x_Si(OR’)_4-x_ [25,26], the pH [27], the proportions of H_2_O/TEOS and EtOH/TEOS [18,26,27], and the curing and drying methods [28,29]. Changes in these factors can produce significant variations in the density and porosity of the resulting xerogel. In this way, the properties of these materials can be controlled by acting on their molecular structure and morphology.

In previous studies, the formation of ordered domains in the amorphous structure of hybrid silicon xerogels was evidenced by: (i) a signal in the X-ray diffraction spectra at 2θ < 10°, (ii) the appearance in the NMR spectra of T^2,3^ structures (T^n^, notation used for silicon atoms bonded to three oxygen atoms and Q^n^, for silicon atoms bonded to four bridged oxygen atoms) [30,31], and (iii) a band at around 1150 cm^−1^ in the Fourier-transform infrared spectroscopy (FTIR) spectra [20,21,22]. These observations are consistent with data obtained by mass spectrometry (MS) [21], simulations of the FTIR spectra, and inelastic neutron spectroscopy (INS). We also constructed a theoretical cage model to explain the presence of ordered domains in these materials and increase knowledge of the properties and the processes involved in their formation [32].

The aim of the present study was to determine the influence of the alkyl chain and chlorine atom on the morphological and textural properties of various hybrid materials produced by co-condensation. For this purpose, three new series of organochlorinated xerogels were prepared and characterized using TEOS and a chloroalkyltriethoxysilane (ClRTEOS; R = methyl [M], ethyl [E], or propyl [P]) at different molar ratios. These chlorinated precursors have been used as silane coupling agents or in the preparation of polyhedral oligomeric silsesquioxanes (POSS) [33,34,35]; however, to our best knowledge, they have not been studied in co-condensation with tetraalkoxysilanes. Development of the structure and morphology of these materials was characterized by ^29^Si NMR (Nuclear Magnetic Resonance), XRD (X-ray Diffraction), FTIR (Fourier-Transform Infrared Spectroscopy), helium pycnometry, FE-SEM (Field-Emission Scanning Electron Microscopy), and N_2_ and CO_2_ adsorption. The influence of the halogen was also explored by comparison with results previously obtained for analogous alkyl xerogels (RTEOS:TEOS) [20,21,22]. The ultimate application of these materials is to use them as coatings for optical fiber sensors, for which a labile and specific interaction with the analyte is necessary, hence the importance of increasing knowledge on their properties to be able to develop siliceous materials with tailored properties and porosities.

## 2. Materials and Methods

Three series of hybrid xerogels were synthesized by the sol-gel method at pH = 4.5 [36] using two precursors at different molar ratios, TEOS:ClMTEOS, TEOS:ClETEOS, and TEOS:ClPTEOS (TEOS = tetraethoxysilane, ClMTEOS = (chloromethyl)triethoxysilane, ClETEOS = (2-chloroethyl)triethoxysilane, and ClPTEOS = (3-chloropropyl)triethoxysilane), maintaining a constant precursor:ethanol:water ratio of 1:4.75:5.5 [27,37].

### 2.1. Materials

Siliceous precursors TEOS (purity > 99%), ClMTEOS (purity > 95%), and ClPTEOS (purity > 95%) were supplied by Sigma-Aldrich (St. Louis, MO, USA), and ClETEOS (purity > 95%) was supplied by Flurochem (Glossop, Derbyshire, UK). Absolute ethanol (GPRA analysis) and HCl were purchased from Merck (Darmstadt, Germany). All of these chemicals were used without further purification.

### 2.2. Synthesis of Silicon Hybrid Xerogels

First, the siliceous precursors TEOS and ClRTEOS (ClMTEOS, ClPTEOS, or ClETEOS) were mixed in a 30 mL container with diameter of 3.5 cm and threaded plastic lid (Scharlab, Barcelona, Spain). Absolute ethanol (as solvent) and Milli-Q grade water were added dropwise under magnetic stirring to facilitate miscibility, using an automatic burette (Tritino mod. 702 SM, Metrohm, Herisau, Switzerland). The solution pH was adjusted to 4.5 with 0.05 M HCl. The containers were placed in a reciprocating shaker (SO1- Bibby Stuart, Stone, UK) and kept in a thermostatized oven at 60 °C (J.P. Selecta S.A., Barcelona, Spain) until gelling. The materials were considered gelled if their shape did not change when the container was tilted.

After gelling, the alcogels were cured with 5 mL of ethanol for 1 week at room temperature with the lid closed. Next, the containers were covered with parafilm™, which was punctured with small holes to facilitate solvent evaporation, and were then dried at room temperature and atmospheric pressure [28]. Some samples developed cracks and fractures during the drying process, attributable to stress generated by capillary forces within the pores [38].

### 2.3. Characterization of Silicon Hybrid Xerogels

The amorphous nature of hybrid xerogels means that numerous techniques must be applied to characterize their structure and properties [39].

^29^Si Cross Polarization Magic-Angle Spinning (CP MAS) solid-state NMR was recorded on a Bruker AV-400 MHz spectrometer (Billerica, MA, USA) operating at 79.5 MHz for ^29^Si. Spectra were obtained at room temperature, giving chemical shifts in parts per million relative to tetramethylsilane, using ^1^H decoupling, a rotation frequency of 5 kHz, and 800 scans per spectrum. The classical notation in ^29^Si NMR studies was employed, i.e., T for silicon atoms bound to three oxygen atoms capable of forming siloxane bridges (from precursors), and Q for silicon atoms bound to four oxygen atoms capable of forming siloxane bridges (from TEOS). T and Q notations were completed with superscript i (T^i^, i = 0, 1, 2 or 3; Q^i^, i = 0, 1, 2, 3 or 4) for the number of Si–O–Si bridges in each silicon atom [40].

X-ray diffraction patterns were obtained at room temperature, using a PANalytical Empyrean XRD instrument (Empyrean, Almelo, The Netherlands) with copper rotating anode and graphite monochromator (at 45 kV and 40 mA) to select the CuK_α1/2_ wavelength at 1.54 nm. Measurements were performed in a stepped scan mode of 2 ≤ 2θ ≤ 50° in steps of 0.013° at a rate of 0.5 steps s^−1^ [41].

N_2_ and CO_2_ adsorption isotherms (at −196 and 0 °C, respectively) were determined with a volumetric adsorption system (ASAP2020, Micromeritics, Norcross, GA, USA), weighing approximately 150 mg of sample into a straight-walled Pyrex glass tube followed by degassing at 150 °C for ≤ 6 h with a residual vacuum of < 0.66 Pa. Analysis time ranged from 14 to 55 h for N_2_ adsorption and from 2.5 to 9 h for CO_2_ adsorption). For N_2_ adsorption, the sample tube was covered with an isothermal jacket and immersed in a Dewar with liquid nitrogen (−196 °C). For CO_2_ adsorption, the tube was placed in a thermostatized recirculation bath (PolyScience, Niles, IL, USA) at 0 °C, using ethylene glycol as a refrigerant. The recorded adsorption data were analyzed with the Microactive (version 4.06) software of the system, adjusting the parameters as appropriate for each model. Specific surface areas were calculated using two techniques: (i) the Brunauer–Emmett–Teller (BET) model (a_BET_) and (ii) the Dubinin-Radushkevich (DR) method, applying a molecular section of 0.17 nm^2^ to CO_2_ (a_DR_) [42]. Pore volumes were defined by their diameter (∅): (i) The volume of micropores (∅ ≤ 2 nm) was obtained from the DR method (V_micro(N2)_ and V_micro(CO2)_), (ii) the volume of mesopores (2 < ∅ ≤ 50 nm) was calculated by abstracting the amount of N_2_ adsorbed at p/p^o^ = 0.3 from that adsorbed at p/p^o^ = 0.8 (V_meso(N2)_), and (iii) the total volume (V_Total(N2)_) was considered as the amount of N_2_ adsorbed at p/p^o^ = 0.95. Liquid densities of the adsorbates were obtained from the literature (0.808 g cm^−3^ for N_2_ and 1.023 g cm^−3^ for CO_2_) [43,44]. Porosity distributions were determined according to density-functional theory (DFT) using SAIEUS software and applying the “carbon-N2-77, 2D-NLDFT heterogeneous Surface” model for N_2_ adsorption and the “carbon-CO2-273, 2D-NLDFT Het Surface, pp max = 10 atm” model for CO_2_ adsorption. Mean pore sizes were further determined by applying the Barrett–Joyner–Halenda (BJH) method to the desorption curves, using a Kruk–Jaroniec–Sayari correction and thickness curve.

Skeletal density was measured using a helium pycnometer (AccuPyc 1330, Micromeritics, Norcross, GA, USA), performing a routine calibration before analyses (20 purges and 10 measurements). The sample was weighed into a 1 cm^3^ cell, which was filled to the maximum possible to reduce measurement inaccuracies caused by dead volumes. Each sample was analyzed using 10 purges and 5 measurements.

Field-emission scanning electron microscopy (FE-SEM) provides high-resolution images of sample surfaces, and energy-dispersive X-ray spectroscopy (EDX) reveals the surface distribution of the atoms. Micrographs were obtained with a Carl Zeiss SMT field emission scanning electron microscope (Carl Zeiss SMT, Oberkochen, Germany), at 200 kV.

Infrared spectra were obtained using a FTIR spectrometer (Jasco mod. 4700, Japan), yielding the following two spectral ranges with two different sample concentrations in order to facilitate band comparisons: (i) 4000–2200 cm^−1^, recorded with 2 mg of sample dispersed in 200 mg KBr to examine in greater detail the region where hydroxyl and C–H bonds appear and (ii) 2200–400 cm^−1^, recorded with 0.6 mg of sample in 200 mg KBr to avoid saturation of the Si–O–Si asymmetric stretching signal [45]. Tablets were dried overnight in an oven at 115 °C under vacuum to minimize the amount of water adsorbed. Spectra were recorded using 25 scans and a resolution of 4 cm^−1^.

## 3. Results

### 3.1. ^29^Si Nuclear Magnetic Resonance (NMR)

**Figure 1:**
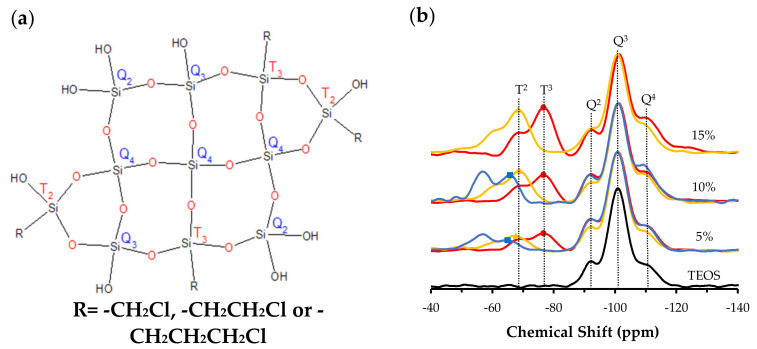
(**a**) Silicon environments present in chloroalkyltriethoxysilane (ClRTEOS):tetraethoxysilane (TEOS) xerogels, and (**b**) normalized ^29^Si NMR spectra of the hybrid xerogels synthesized with 5%, 10%, and 15% of ClRTEOS (chloromethyltriethoxysilane (ClMTEOS) (●), chloroethyltriethoxysilane (ClETEOS) (▲), and chloropropyltriethoxysilane (ClPTEOS) (■)).

^29^Si NMR spectra were obtained to study the effect of chloroalkyl precursors on the relative abundance of silicon species in hybrid xerogels. In all cases, they showed the characteristic bands of the different species of silicon that can be found in this type of material, represented in Figure 1a.

Figure 1b depicts ^29^Si NMR spectra for the three series of ClRTEOS:TEOS xerogels, normalized with respect to the Q^3^ signal, which was always the most intense signal and corresponds to the dominant species in these materials synthesized in acid media. Q^1^ and T^1^ signals are not detected [20], and the proportions of T^2^ and T^3^ species vary according to the precursors. Thus, T^3^ species are more abundant than T^2^ species at all molar percentages in xerogels prepared with ClMTEOS and ClETEOS, indicating that these xerogels are preferably formed by the most condensed species. Spectra for the series prepared with ClPTEOS show a predominance of T^2^ species, implying that more silicon atoms are partially condensed and attached to hydroxyl groups. Spectra for the Q species reveal a decrease in Q^3^ and Q^2^ species with an increase in the molar percentage of chlorinated precursor.

**Figure 2:**
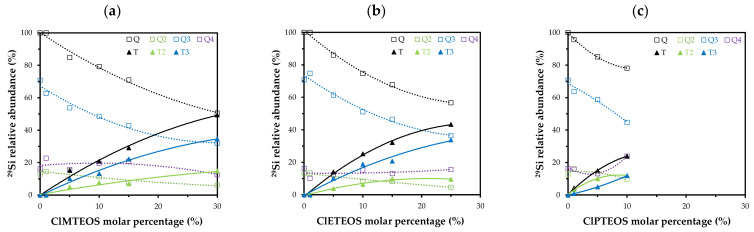
Variation in the relative abundance of the condensed species of Si with respect to percentage ClRTEOS obtained by integrating the ^29^Si NMR spectra for: (**a**) ClMTEOS, (**b**) ClETEOS, and (**c**) ClPTEOS.

Figure 2 depicts the relative abundance of species detected in the spectra for the three series of hybrid xerogels ClRTEOS:TEOS. It shows the time course of the total proportion of Q (Q^2^ + Q^3^ + Q^4^) and T (T^2^ + T^3^) species as a function of the percentage of ClRTEOS and the time course of each individual species. It can be observed that Q^2^ and Q^3^ species always decrease with increased molar percentage of the chlorinated precursor, while the results for Q^4^ differ according to the precursor used, with a lower abundance in the ClMTEOS series and a slightly higher abundance in the ClPTEOS series. Figure 2 also shows that T^3^ species predominate over T^2^ species in the ClMTEOS and ClETEOS series, whereas T^2^ species predominate in the ClPTEOS series. This is attributable to the steric hindrance exerted by the chloropropyl chain in the condensation reactions, hampering complete condensation around the silicon atoms. The chloropropyl chain also influences the surface charge of the colloids and generates repulsion between them, consistent with the exponential increase in gelation times of this series at the highest molar percentages (Table S1).

Chen et al. reported that T^3^ and Q^4^ species are predominant in POSS synthesized with ClMTEOS [35]. In the present study, T^3^ species are associated with rings formed by cyclic tetramers of (SiO)_4_ that build T_8_ cages, while Q^4^ would be the silicon atoms at the vertex that bind T_8_ structures together. The minor signal detected in the POSS, T^2^, is related to the presence of partially open cages (T_7_). The proportion of Q species has also been associated with the FTIR spectra, with reports that Q^2^, Q^3^, and Q^4^ species present their asymmetric Si–O–Si stretching vibration mode at different frequencies (1000–1030, 1100, and 1150–1200 cm^−1^, respectively) [46,47] and that the aforementioned polycyclic structures ((SiO)_4_ and T_8_ cages) are related to bands at 1090 and 1150 cm^−1^, respectively [48]. It can therefore be concluded that the presence of these structures in the materials is consistent with the increase in T^3^, Q^3^, and Q^4^ species when the amount of precursor is larger and with the shoulder at 1150 cm^−1^ observed in the FTIR spectra (Figure S1).

Table 1 exhibits the chemical shifts for the different silicon atoms of each hybrid xerogel, showing that T and Q signals are not affected in any series by the increase in molar percentage of ClRTEOS or RTEOS, because there is no significant change in the silicon environment. However, a marked shift in T signals is observed in the comparison of different series with the same percentage of precursor. This is attributable to a shielding effect in which the organic precursor acts by adding or removing charge density to the silicon atom, thereby shifting the signal to a higher or lower field [49,50]. The chlorine atom removes charge density, reducing the donor capacity of the alkyl chains and increasing the positive charge of the silicon atom, and this effect is reflected in the shift of its signal to lower ppm. Likewise, the further the chlorine is from the silicon atom, the lesser is the shift of the signal. This trend is readily observed by comparing the average T^3^ chemical shift between the chloroalkyl and the alkyl series: (i) −12.2 ppm when chlorine is in carbon α position (MTEOS vs. ClMTEOS), (ii) −4.4 ppm when it is in β position (ETEOS vs. ClETEOS), and (iii) −0.45 ppm when it is in γ position (PTEOS vs. ClPTEOS).

### 3.2. X-Ray Diffraction (XRD)

**Figure 3:**
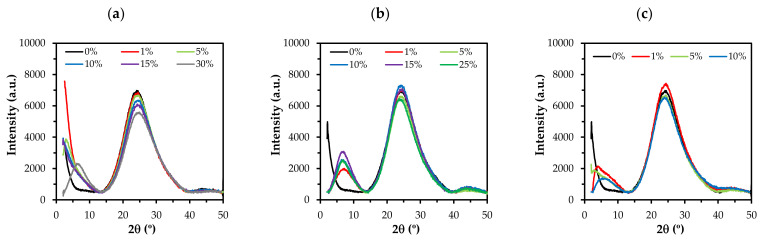
X-ray diffraction patterns of the hybrid xerogels ClRTEOS:TEOS at different molar percentages: (**a**) ClMTEOS, (**b**) ClETEOS, and (**c**) ClPTEOS.

Figure 3 displays the XRD spectra of the xerogels synthesized with ClMTEOS, ClETEOS, and ClPTEOS. The diffraction pattern shows a broad signal at around 2θ = 24°, characteristic of amorphous silica. This signal corresponds to the distance between silicon atoms connected by siloxane bonds [49]. It can be observed that the intensity of this signal is reduced with an increased molar percentage of the precursor and that another signal arises with higher molar percentage of ClMTEOS and ClPTEOS (30% and 10%, respectively) at 2θ < 10°. The behavior of the ClETEOS series is different, observing the presence of this signal at the lowest molar percentage (1%) and an increase in its intensity with a larger amount of precursor. The 2θ < 10° signal is associated with the formation of ordered structures composed of T_8_ cages or ladders, which are, in turn, related to lamellar morphologies [51,52,53]. The distances calculated from this maximum correspond to the distance between the organic substituents in these structures [32]. The three series show this band at lower molar percentages in comparison to those previously observed for their alkyl equivalents, being detected in 70% of MTEOS, 30% of ETEOS, and 10% of PTEOS series [20,21,22]. This indicates a higher tendency for the chloroalkyl precursors to form ordered domains in the amorphous matrix. There is also a broad low-intensity signal at 2θ ~ 45°. According to Bragg’s law, these bands correspond to replicas of the signal at 2θ = 24°, which supports the presence of local periodicity in the silica matrix.

Table 2 displays the angles of each signal (2θ), the area of the peaks, and the Si−O−Si bond distances. It shows the shift of the amorphous silica signal (2θ ~ 24°) as a function of the precursor and its molar percentage. There is a shift to larger angles in the ClMTEOS series, which results in a shortening of the Si−O−Si bond distance (0.368 nm for 0% and 0.359 nm for 30%), whereas there is a shift to smaller angles in the ClETEOS and ClPTEOS series, resulting in longer bond distances (0.372 nm for 25% ClETEOS and 0.370 nm for 10% ClPTEOS). These modifications of bond distances are attributable to inductive and steric effects generated by the chloroalkyl substituents of the precursors. The shorter bond distances for the ClMTEOS series result from the predominance of the inductive effect of the chlorine atom, which removes electron density from the silicon atom and therefore polarizes the adjacent Si−O bond. On the contrary, in ClETEOS and ClPTEOS series, the effect of the alkyl chain predominates over the inductive effect of the chlorine atom, thus elongating the adjacent Si−O bond as occurred with their non-chlorinated analogues.

### 3.3. Skeletal Density

**Figure 4:**
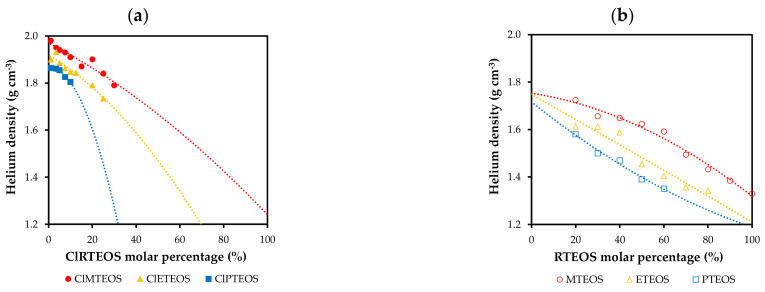
Skeletal density of the materials according to the precursor molar percentage for: (**a**) ClRTEOS series (ClMTEOS, ClETEOS, and ClPTEOS) and (**b**) RTEOS series (MTEOS, ETEOS, and PTEOS) in previous studies [20,21,22]. Reference material density (100%TEOS) = 1.96 g·cm^−3^.

Helium pycnometry reveals the skeletal density of the synthesized xerogels. Figure 4 depicts the variation in skeletal density as a function of the molar percentage of the precursor for the chloroalkyl series (Figure 4a) and for the analogous alkyl series prepared in previous studies (Figure 4b). The density of the chloroalkyl xerogels decreases with a greater proportion of the precursor, because the precursor blocks one of the hydrolysis and condensation positions, reducing the degree of cross-linking. Another influencing factor is the nature of the substituent, observing a lower skeletal density with longer alkyl chain due to greater steric hindrance. In the previously studied alkyl xerogels, the XRD spectra show an elongation of the siloxane bonds consistent with the donor effect of the carbon atom directly bound to silicon, leading to lesser compaction and therefore, lower skeletal density [20,21,22]. In the chloroalkyl series, densities are higher than those of their non-chlorinated analogues at the same molar percentages due to the attractor effect of chlorine. For example, at a molar percentage of 10%, the density value is 1.91 g·cm^−3^ for the ClMTEOS xerogel vs. 1.7 g·cm^−3^ for the MTEOS xerogel.

### 3.4. Porous Texture

#### 3.4.1. Adsorption Isotherms and Textural Properties

**Figure 5:**
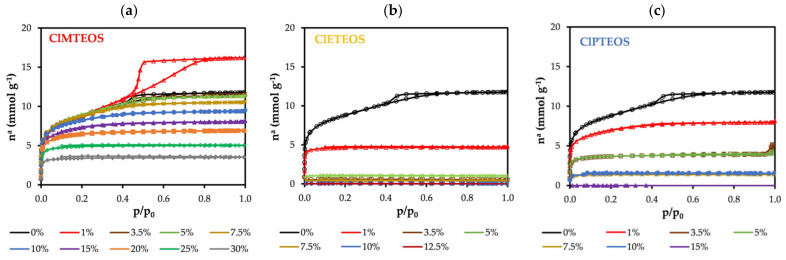
N_2_ isotherms (−196 °C) of chloroalkyl materials at different molar percentages of: (**a**) ClMTEOS, (**b**) ClETEOS, and (**c**) ClPTEOS.

The nitrogen adsorption isotherms of the ClRTEOS:TEOS series are exhibited in Figure 5. The isotherm of the reference material has a flat knee (Type I(b) isotherm), an adsorption branch with a positive slope, and a hysteresis loop in the desorption branch (H2 (a)), typical of type IV(a) isotherms. It is therefore a material with a mixed Type I(b)-IV(a) isotherm, indicating micro-mesoporosity [54].

With respect to the reference material, the isotherms of the hybrid xerogels prepared in this study show that they adsorbed a smaller amount of N_2_, gradually becoming more microporous. The knees are sharper, the plateaus are practically parallel to the abscissa axis, and the hysteresis loop disappears. Figure 5 depicts the N_2_ isotherms of the three series of chloroalkyl xerogels. In the ClMTEOS series (Figure 1a), Type IV isotherms become Type I(b) and I(a) with increased molar percentage of the precursor (10% and 30%, respectively), and N_2_ adsorption ceases when the highest molar percentage is reached (35%) (Table 3). In contrast, Figure 5a depicts the striking observation of a Type IV(a) isotherm for the material containing 1% of ClMTEOS, showing a similar knee but wider hysteresis loop in comparison to the reference material (Type H1). Dudás et al. prepared hybrid xerogels with low proportions of MTEOS and also observed increased N_2_ adsorption, associated with the transition from cone to ink-bottle shaped pores that is responsible for the Type H1 hysteresis loop [55]. The ClETEOS series (Figure 5b) has a type I(a) isotherm at a precursor molar percentage of 1%, whereas N_2_ adsorption is very low at higher percentages and ceases at 12.5%. The CIPTEOS series (Figure 5c) changes from Type I(b) at a percentage of 1% to Type I(a) at percentages up to 10%, with a cessation of adsorption at higher percentages, as in the case of the ClETEOS xerogels. The isotherms in Figure 5 are depicted on a semi-logarithmic scale in Figure S2 (Supplementary Materials) to visualize more clearly the development of the microporosity at higher molar percentages of the precursor. Hence, the incorporation of chlorinated precursors into the structure of TEOS modifies its porosity, reducing the size, volume, and shape of the pores in the following order: ClETEOS > ClPTEOS > ClMTEOS.

**Figure 6:**
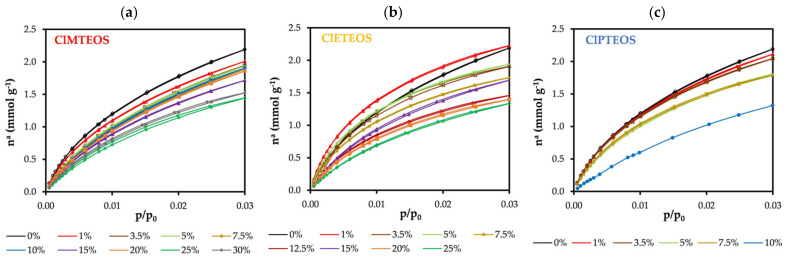
CO_2_ isotherms (0 °C) of chloroalkyl materials at different molar percentages of: (**a**) ClMTEOS, (**b**) ClETEOS, and (**c**) ClPTEOS.

At least two adsorbates are required to characterize the porosity of a material. N_2_ adsorption (−196 °C) has long been complemented by CO_2_ adsorption (0 °C), allowing differentiation between microporosity below 0.7 nm and that above 0.7 nm (associated with cooperative processes of the adsorbate molecules). The isotherms displayed in Figure 6 show a similar trend for all of the materials, evidencing a lesser adsorption of CO_2_ at higher molar percentages of the precursor.

Table 3 displays the textural values calculated from N_2_ and CO_2_ isotherms. A reduction in the specific surface areas a_BET_ (N_2_) and a_DR_ (CO_2_) can be observed with a higher percentage of ClRTEOS. The pore volume data confirm the essentially microporous nature of the material, given that V_micro_(N_2_) > V_meso_(N_2_), and V_meso_ tend towards zero with a higher percentage of the precursor. Notably, the material becomes ultramicroporous at ClETEOS molar percentages above 1%, explaining the lack of N_2_ adsorption. Higher and more constant adsorption energies (calculated by DR method) were observed for CO_2_ than for N_2_, consistent with kinetic restrictions of N_2_ entry into the narrowest pores. It should be noted that the kinetic energy of CO_2_ is lower than that of N_2_ at the higher molar percentages of ClMTEOS (20% and 25%) and the lowest ratio of ClETEOS (1%). This is because the N_2_ retention mechanism within the narrow micropores (r_pores_/r_adsorbate_ ~ 1.1) is based on the overlap of pore wall potentials (∅ < 0.7 nm). This behavior was also described in analogous non-chlorinated xerogels [56].

#### 3.4.2. Porosity Distribution

Two distinct theoretical approaches were adopted to explore the effect on porosity of the precursors. The BJH method was used to calculate the mean pore size from the desorption branch of the isotherms. Application of this method showed a reduction in mean pore size proportional to the precursor molar percentage (Table 3), from 4.21 to 2.09 nm of ClMTEOS and from 3.39 to 2.18 nm of ClPTEOS. In the ClETEOS series, N_2_ adsorption is only observed at a molar percentage of 1%, and this material has a value of 2.32 nm.

**Figure 7:**
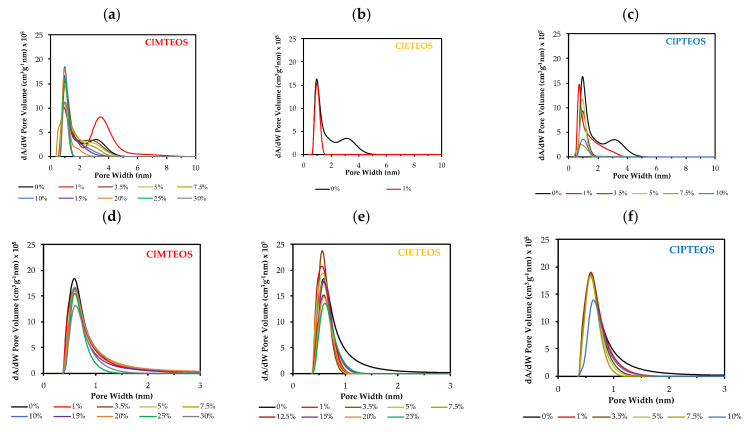
Density-functional theory (DFT) porosity distribution obtained from N_2_ isotherms of: (**a**) ClMTEOS:TEOS series, (**b**) ClETEOS:TEOS series, and (**c**) ClPTEOS:TEOS series and from CO_2_ isotherms of: (**d**) ClMTEOS:TEOS series, (**e**) ClETEOS:TEOS series, and (**f**) ClPTEOS:TEOS series.

The DFT was also applied to calculate porosity distributions from N_2_ and CO_2_ adsorption data (Figure 7a–f, respectively), showing a similar influence of the precursor in both cases. Thus, N_2_ isotherm data show a progressive narrowing of the pores and a loss of mesoporosity with a higher percentage of precursor, and the CO_2_ isotherm data also show a sharpening of the micropore distribution. Note should be taken of the aforementioned irregular behaviors of: (i) 1% ClMTEOS material (Figure 7a), which has an increased pore size in comparison to the reference material, becoming more mesoporous, and (ii) the ClETEOS series (Figure 7b), whose ultramicroporous nature means that pore distribution values can only be obtained for the material with a molar percentage of 1%, observing a gradual loss of porosity with higher percentages of the precursor (Figure 7e).

### 3.5. Field-Emission Scanning Electron Microscopy (FE-SEM)

**Figure 8:**
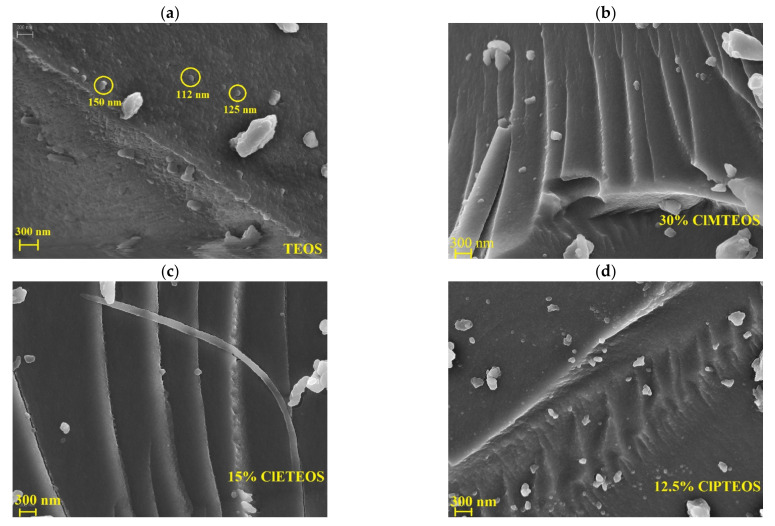
FE-SEM micrographs of: (**a**) reference xerogel (100% TEOS), (**b**) xerogel synthesized with 30% of ClMTEOS, (**c**) xerogel synthesized with 15% ClETEOS, and (**d**) xerogels synthesized with 12.5% ClPTEOS.

Figure 8 depicts FE-SEM micrographs of the reference material and a sample of each series synthesized with chloroalkyl precursors (see Figure S3 for micrographs at lower molar percentages). The surface of samples in Figure 8 was studied with energy-dispersive X-ray spectroscopy (EDX) to confirm that the chlorine atoms of the chloroalkyl precursors are homogeneously distributed on their surface (Figure S4).

The reference material (Figure 8a) has a rough surface made up of globular particles that melt, giving it a scaly appearance. The particle diameter ranges from 110 to 150 nm, and the inter-particle spacing correlates with the narrow mesoporosity recorded by the isotherm [57]. The surface of the xerogel synthesized with 1% ClMTEOS (Figure S3a) is also formed by globular particles, which are smaller and difficult to detect. This morphological change results from both the increase in pore volume and the transition from cone to ink-bottle shaped pores. The surface particles fade with increased molar percentage of ClMTEOS, which gives rise to thin layers that overlap and intersect, yielding a highly microporous and compact material in accordance with its isotherm (Type I(a)).

Micrographs of the ClETEOS series (Figure 8c and Figure S3d–f) show smooth flake-like surfaces consistent with the lack of N_2_ adsorption of this xerogels. The same pattern is observed in the ClPTEOS series (Figure 8d and Figure S3g–i), although a larger amount of precursor is needed to bring about these changes.

In all three ClRTEOS series, the particle size decreases with higher percentages of the precursor. This effect is associated with the lower degree of triethoxysilane cross-linking in comparison to TEOS and with the nanostructuration generated to minimize the repulsion between colloids caused by the surface chloroalkyl groups. The decrease in particle size results in the formation of compact but less dense sheets that are responsible for the lower mesoporosity and lesser N_2_ adsorption of these materials.

## 4. Conclusions

Hybrid ClRTEOS:TEOS xerogels were prepared at pH = 4.5 up to molar percentages of 35% (ClMTEOS) and 25% (ClETEOS and ClPTEOS). Signals of triethoxysilanes (T^n^) in ^29^Si NMR spectra confirm the presence of the chloroalkyl substituents in the silicon matrix, indicating the stability of Si–C bonds under the synthesis conditions used. These signals are mainly found for the most condensed species (T^3^) in the ClMTEOS and ClETEOS series, observing a greater proportion of condensed species of TEOS, Q^3^, and Q^4^, with a higher percentage of the precursor. This predominance of the most condensed species is consistent with the presence of a signal at a small angle (2θ < 10°) in the XRD spectra and with the shortening of Si–O–Si bonds compared with their non-chlorinated analogues, indicating the presence of ordered domains constituted by tight and compact structures, such as short ladders and T_8_ cages. The three series of xerogels show higher skeletal densities in comparison to their analogous non-chlorinated series due to the electronic and steric effects of the chloroalkyl precursor. With regard to the texture of the materials, FE-SEM micrographs show changes in their surface morphology from a granular to a smoother and more compact texture with higher precursor percentages. These modifications also reflect the decrease in N_2_ and CO_2_ adsorption capacity recorded by the isotherms with a higher percentage of precursor, allowing materials with different pore sizes to be tailored. XRD spectra show that the chloroalkyl moiety of the precursors more efficiently promotes the ordering of the local structure in comparison to the alkyl chain in their non-chlorinated analogues. Strikingly, a very small amount of precursor (1% molar percentage) is required in the ClETEOS series to induce periodic domains formed by well-defined ladders and T_8_ cage-like structures, producing a significant change in both structural and textural properties with respect to the reference material. The synthesis of these new locally nanostructured hybrid materials with tunable porosities opens up a very promising path for their use in a wide range of applications such as membranes, conductive films, absorbents, catalysis, optoelectronics, and coatings for optical sensors.

## Figures and Tables

**Table 1:** Signals from the ^29^Si NMR spectra of the hybrid xerogels chloroalkyltriethoxysilane (ClRTEOS):tetraethoxysilane (TEOS) and alkyltriethoxysilane (RTEOS): tetraethoxysilane (TEOS) (R = methyl, ethyl, and propyl) [20,21,22].

Precursor	Precursor Molar Percentage (%)	^29^Si RMN (ppm)				
		T^2^	T^3^	Q^2^	Q^3^	Q^4^
**ClMTEOS**	5	−68.5	−76.6	−92	−100.9	−110.4
	10	−68.9	−76.8	−91.8	−101.0	−110.1
	15	−68.3	−76.7	−92.9	−101.3	−110.4
**ClMTEOS**	5	−60.2	−67.7	−91.7	−100.8	−109.7
	10	−60.3	−68.8	−91.7	−100.8	−109.7
	15	−60.3	−68.7	−91.9	−100.9	−109.5
**ClPTEOS**	5	−57.0	−65.0	−91.6	−100.8	−110.3
	10	−57.4	−65.5	−91.9	−100.7	-109.6
**MTEOS**	30	−54.8	−63.1	−91.7	−101.1	−110.9
	70	−56.3	−64.6	_a_	−100.9	−109.6
	100	−57.0	−65.8	_a_	_a_	_a_
**ETEOS**	10	−54.6	−63.2	−92.5	−101.7	−110.7
	30	−55.7	−63.8	−92.3	−101.3	−109.9
	60	−56.4	−65.0	_a_	−101.9	−110.0
**PTEOS**	10	−56.4	−64.1	−92.2	−100.9	−110.2
	30	−56.1	−64.6	−90.9	−100.3	−109.4
	60	−56.8	−65.7	_a_	−101.4	−110.4

_a_—Non detected.

**Table 2:** Bragg angles (2θ), band area (A), and bond distance (d1 and d2 (nm)) calculated from the XRD bands of the xerogels synthesized with different precursors and molar percentages.

Precursor	ClRTEOS	Peak 2θ < 10°			Peak 10° < 2θ < 30°		
	Molar Percentage (%)	2θ_1_ (°)	A_1_	d_1_ (nm)	2θ_2_ (°)	A_2_	d_2_ (nm)
**TEOS**	0	_a_	_a_	_a_	24.16	68,812	0.368
**ClMTEOS**	1	_a_	_a_	_a_	24.26	66,451	0.367
	5	_a_	_a_	_a_	24.30	65,847	0.366
	10	_a_	_a_	_a_	24.54	62,627	0.363
	15	_a_	_a_	_a_	24.42	60,377	0.365
	30	6.52	10,217	1.35	24.78	56,217	0.359
**ClMTEOS**	1	7.17	8594	1.23	24.56	68,577	0.362
	5	6.92	15,879	1.28	24.30	74,258	0.366
	10	6.76	16,568	1.31	24.14	81,785	0.369
	15	6.76	19,509	1.31	24.22	78,157	0.367
	25	6.76	15,780	1.31	23.90	71,722	0.372
**ClPTEOS**	1	_a_	_a_	_a_	24.22	73,819	0.367
	5	_a_	_a_	_a_	23.98	65,782	0.371
	10	5.80	5514	1.52	24.06	66,143	0.370

_a_—Non detected.

**Table 3:** Textural parameters of chloroalkyl materials at different molar percentages.

Precursor	ClRTEOS Molar Percentage (%)	a_BET_ (N_2_)	a_DR_ (CO_2_)	V_micro_ (N_2_)	V_micro_ (CO_2_)	V_meso_ (N_2_)	V_total_ (N_2_)	BJH APS ^a^	E_c_ (N_2_) ^b^	E_c_ (CO_2_) ^b^
		(m^2^ g^−1^)		(cm^3^ g^−1^)				(nm)	(KJ mol^−1^)	
**TEOS**	0	697	510	0.283	0.195	0.074	0.407	3.61	15.27	19.71
**ClMTEOS**	1	700	465	0.289	0.178	0.210	0.560	4.21	14.27	19.77
	3.5	691	475	0.285	0.182	0.061	0.394	3.55	14.82	18.93
	5	702	464	0.293	0.177	0.052	0.390	3.51	14.93	19.15
	7.5	693	457	0.288	0.175	0.036	0.364	3.41	15.26	18.98
	10	662	471	0.274	0.180	0.022	0.324	3.38	15.67	18.77
	15	591	428	0.248	0.164	0.013	0.278	3.30	15.81	18.82
	20	534	463	0.226	0.177	0.009	0.239	3.15	16.42	18.71
	25	422	381	0.175	0.146	0.002	0.174	2.56	18.32	18.29
	30	294	358	0.121	0.137	0.003	0.123	2.09	20.47	18.32
	35	_c_	347	_c_	0.132	_c_	_c_	_c_	_c_	18.04
**ClMTEOS**	1	410	506	0.164	0.193	0.001	0.164	2.32	22.17	20.80
	3.5	_c_	461	_c_	0.178	_c_	_c_	_c_	_c_	20.05
	5	_c_	451	_c_	0.172	_c_	_c_	_c_	_c_	20.64
	7.5	_c_	411	_c_	0.157	_c_	_c_	_c_	_c_	20.25
	10	_c_	371	_c_	0.142	_c_	_c_	_c_	_c_	_d_
	12.5	_c_	356	_c_	0.136	_c_	_c_	_c_	_c_	19.69
	15	_c_	428	_c_	0.164	_c_	_c_	_c_	_c_	19.05
	20	_c_	351	_c_	0.134	_c_	_c_	_c_	_c_	19.29
	25	_c_	345	_c_	0.132	_c_	_c_	_c_	_c_	18.55
**ClPTEOS**	1	555	496	0.232	0.189	0.018	0.282	3.39	16.50	19.74
	3.5	312	483	0.129	0.184	0.004	0.136	2.96	18.48	19.80
	5	318	434	0.129	0.167	0.004	0.136	2.85	19.98	19.49
	7.5	118	433	0.050	0.165	0.000	0.048	2.09	17.37	19.72
	10	132	359	0.056	0.137	0.000	0.053	2.18	15.72	17.69

^a^ Average Pore Size; ^b^ Characteristic energy from Dubinin-raduskevich; _c_ The samples did not adsorb N_2_; _d_ Not calculated

## Data Availability

The data presented in this study are available on request from the corresponding author.

## Supplementary Materials

The following are available online at https://www.mdpi.com/article/10.3390/polym13091415/s1, Table S1: Gelation times of chloroalkyltriethoxysilane (ClRTEOS):tetraethoxysilane (TEOS) materials. Figure S1: FTIR spectra of the xerogels synthesized with 15% precursor in the ranges: (a) 1600–400 cm^−1^ and (b) 4000–2750 cm^−1^; Figure S2: N_2_ adsorption isotherms at −196 °C on a semi-logarithmic scale of the reference material and the hybrids: (a) chloromethyltriethoxysilane (ClMTEOS):TEOS, (b) chloroethyltriethoxysilane (ClETEOS):TEOS, and (c) chloropropyltriethoxysilane (ClPTEOS):TEOS; Figure S3: FE-SEM micrographs of (a) reference material (b–e) ClMTEOS:TEOS materials, (f–i) ClETEOS:TEOS materials, and (j–m) ClPTEOS:TEOS materials; Figure S4: SEM micrographs of the chloroalkyl materials: (a–c) Distributions of chlorine atoms on the surface of xerogels obtained by applying energy-dispersive X-ray spectroscopy (EDX), (d–f) Sum of the EDX spectra obtained by analyzing different points of the micrographs (g–i).
