# Supplementary material for: Novel Organochlorinated Xerogels: From Microporous Materials to Ordered Domains
Source: Polymers (Basel). 2021 Apr 27;13(9):1415. doi: 10.3390/polym13091415 (PMC8123792; doi:10.3390/polym13091415)
Supplement: Supplementary file 1 [file polymers-13-01415-s001.zip › polymers-1197509-supplementary.pdf]

polymers

MDPI

SUPPLEMENTARY MATERIALS

Novel Organochlorinated Xerogels: from Microporous Materials to Ordered Domains

Table S1. Gelation time of ClRTEOS:TEOS materials

RTEOS	RTEOS (%)	t_g (h)
ClMTEOS	5	6
	15	8
	25	11
ClETEOS	5	141
	15	357
	25	815
ClPTEOS	5	13
	15	1199
	25	4769

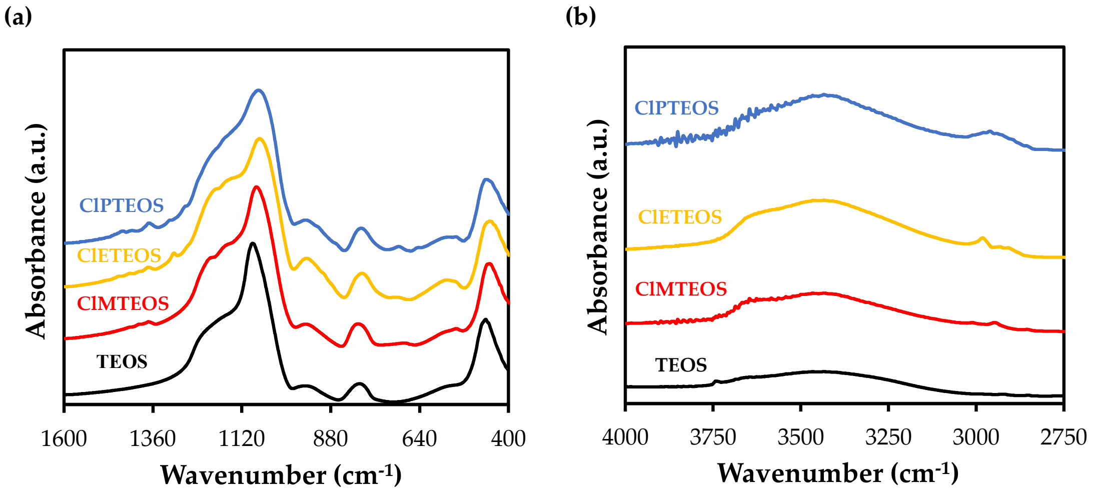

Figure S1. FTIR spectra of xerogels synthesised with 15% precursor within the range of (a) 1600-400 cm^{-1} and (b) 4000-2750 cm^{-1}

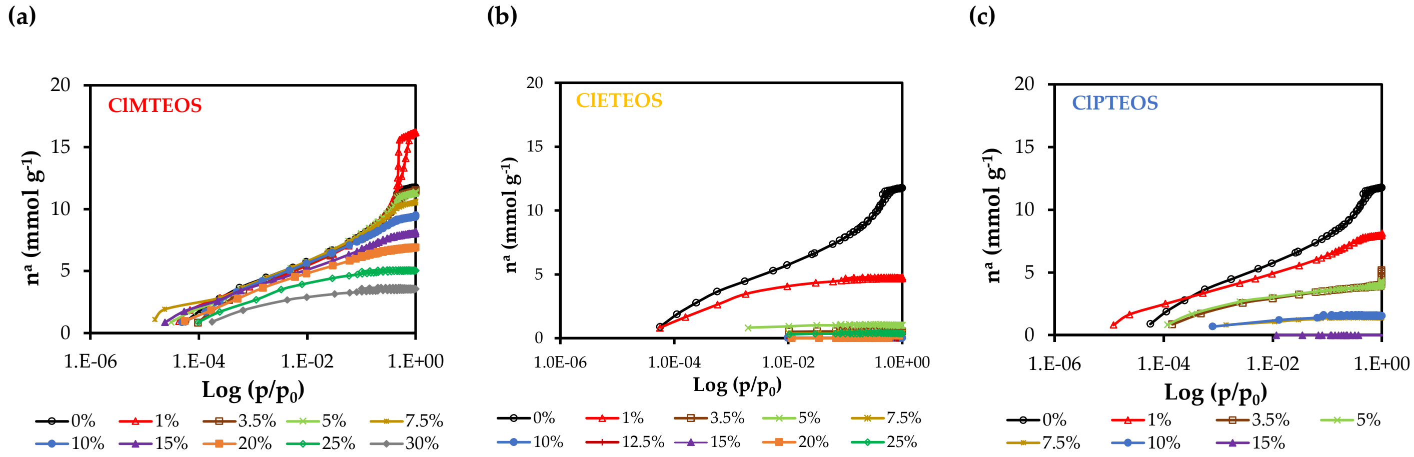

Figure S2. N_2 adsorption isotherms at -196 ºC on a semi-logarithmic scale of the reference material and hybrids (a) ClMTEOS:TEOS, (b) ClETEOS:TEOS, and (c) ClPTEOS:TEOS

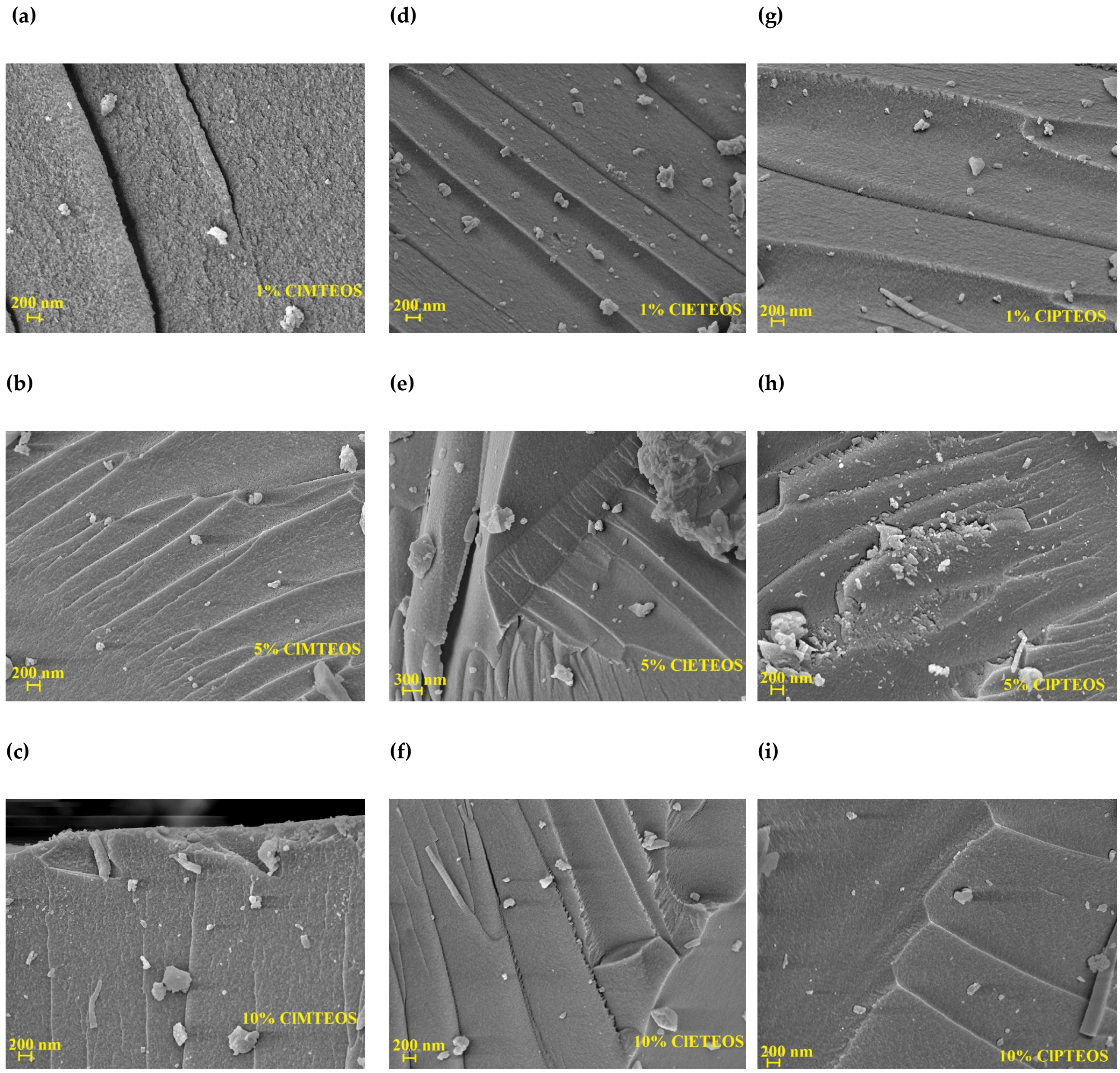

Figure S3. FE-SEM micrographs of (a-c) ClMTEOS:TEOS materials, (d-f) ClETEOS:TEOS materials, and (g-i) ClPTEOS:TEOS materials

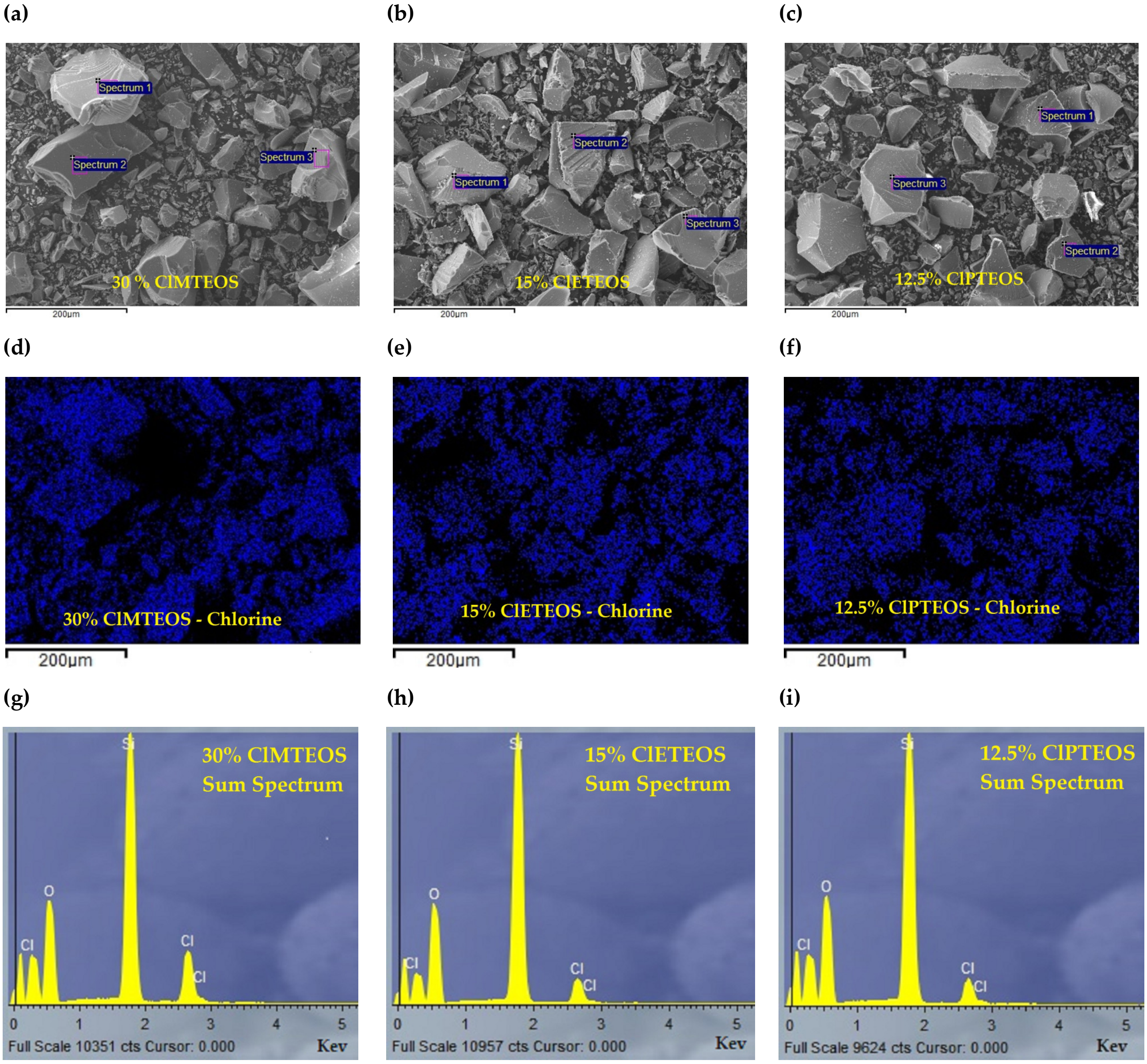

Figure S4. SEM micrographs of the chlroalkyl materials, (a-c) Distribution of chlorine atoms on the surface of xerogels obtained by applying EDX, (d-f) Sum of EDX spectra obtained by analyzing different points of the micrographs (g-i)
